# Gene Expression Profiling of Mycosis Fungoides in Early and Tumor Stage—A Proof-of-Concept Study Using Laser Capture/Single Cell Microdissection and NanoString Analysis

**DOI:** 10.3390/cells10113190

**Published:** 2021-11-16

**Authors:** Justine Lai, Jing Li, Robert Gniadecki, Raymond Lai

**Affiliations:** 1 Department of Medicine, University of Alberta, Edmonton, AB T6G 2R3, Canada; jlai@ualberta.ca; 2 Department of Laboratory Medicine and Pathology, University of Alberta, Edmonton, AB T6G 2R3, Canada; jing070822@163.com; 3 Department of Oncology, University of Alberta, Edmonton, AB T6G 2R3, Canada

**Keywords:** mycosis fungoides, single cell microdissection, NanoString, gene expression profiling, immunohistochemistry

## Abstract

A subset of patients with mycosis fungoides (MF) progress to the tumor stage, which correlates with a worse clinical outcome. The molecular events driving this progression are not well-understood. To identify the key molecular drivers, we performed gene expression profiling (GEP) using NanoString. Ten formalin-fixed/paraffin-embedded skin biopsies from six patients (six non-tumor and four tumor MF) were included; non-tumor and tumor samples were available in three patients. Laser capture/single cell microdissection of epidermotropic MF cells was used for non-tumor cases. We found that the RNA extracted from 700–800 single cells was consistently sufficient for GEP, provided that multiplexed target enrichment amplification was used. An un-supervised/hierarchical analysis revealed clustering of non-tumor and tumor cases. Many of the most upregulated or downregulated genes are implicated in the PI3K, RAS, cell cycle/apoptosis and MAPK pathways. Two of the targets, HMGA1 and PTPN11 (encodes SHP2), were validated using immunohistochemistry. HMGA1 was positive in six out of six non-tumor MF samples and negative in five out of five tumor MF samples. An opposite pattern was seen with SHP2. Our study has provided a proof-of-concept that single-cell microdissection/GEP can be applied to archival tissues. Some of our identified gene targets might be key drivers of the disease progression of MF.

## 1. Introduction

Mycosis fungoides (MF), the most common form of primary cutaneous lymphoid malignancy, is typically characterized by an infiltration of neoplastic CD4-positive T lymphocytes in the skin [1]. The histology of the early-stage skin lesions is variable, but one of the most consistent features is that of epidermotropism of cytologically atypical lymphoid cells with or without the formation of Pautrier microabscesses. With progression to the tumor stage (i.e., ≥1 cm in diameter), epidermotropism often diminishes and the dermal infiltrates of MF cells become more diffuse and confluent. Large cell transformation, defined by the presence of >25% large cells in the infiltrate, occurs mostly in the tumor stage [2]. While MF is generally an indolent disease, progression to the tumor stage elevates the clinical stage of the disease, which is the single most important prognostic factor [1,3,4]. The molecular events underlying this disease progression of MF have not been extensively studied. Currently, there are no biomarkers that are predictive of progression to the tumor stage. 

Gene expression profiling (GEP) has been highly valuable in deciphering the molecular events that drive oncogenesis and cancer progression [5,6,7]. In the literature, we identified only a relatively small number of GEP studies of MF. For instance, Hashikawa et al. employed GEP to identify genes that are differentially expressed between the epidermal and dermal compartments of MF-involved skin, in order to reveal the molecular mechanisms underlying epidermotropism [8]. In another GEP study, Zhang et al. identified genes that are differentially expressed between early-stage MF and benign dermatosis/normal skin, and their results suggest that the differential expression of TOX, PDCD1 and IL23R between the two groups might be useful diagnostically [9]. One of the intrinsic challenges of using GEP to study MF is that MF cells are often surrounded by a relative abundance of benign lymphocytes, stromal cells and epithelial cells. Thus, GEP studies using whole skin biopsies or microdissected regions of a biopsy can potentially produce results that are obscured by signals derived from non-MF cells. This challenge is further compounded by the fact that the MF cell population may represent only a small fraction of the cellular elements present in the entire biopsy sample.

In this study, our main objective was to identify genes that are differentially expressed between non-tumor and tumor MF, with the hypothesis that the identified gene lists may contain key oncogenic drivers for the progression of MF to the tumor stage. To avoid contamination from non-MF cells in the skin biopsy samples, we employed laser capture single-cell microdissection to collect MF cells localized to the epidermis in non-tumor MF cases. Our studies have provided a proof-of-concept that RNA samples extracted from single-cell microdissection of archival tissues are sufficient for GEP using NanoString.

## 2. Materials and Methods

### 2.1. Patient Samples

Formalin-fixed/paraffin-embedded (FFPE) tissue blocks derived from 15 MF skin biopsy samples from 9 patients were retrieved from the Department of Laboratory Medicine and Pathology, University of Alberta. The characteristics of the 9 patients are summarized in Supplementary Table S1. The diagnoses fall into two groups: (1) non-tumor MF (i.e., patch or plaque stage) or (2) tumor MF. Ten samples were used for NanoString analysis and an additional five samples were included for immunohistochemical validation. Patient samples were collected and used in accordance with the ethics guidelines of the Health Research Ethics Board of Alberta (HREBA.CC-16-0859). 

### 2.2. Tissue Staining and Laser Capture Single-Cell Microdissection

FFPE tissues were cut into 4 µm sections, which were then mounted onto a MembraneSlide 1.0 PEN (ZEISS, Oberkochen, Germany). The slides were dipped in wax and stored at −80 °C until being used. Prior to the laser capture single-cell microdissection, the slides were baked for one hour at 60 °C, deparaffinized with xylene and graded alcohols and stained with Harris Hematoxylin. The slides were rinsed with tap water for three minutes and then air-dried. The PALM Microbeam Laser-Capture Microdissection system (ZEISS, Oberkochen, Germany) was employed to collect single MF cells. In non-tumor MF cases, only lymphoid cells in the epidermis (i.e., epidermotropic lymphoid cells) were selected. In tumor cases, single MF cells in areas with diffuse dermal lymphoid infiltration were collected. 

### 2.3. RNA Extraction and Purification

RNA was extracted from the microdissected samples using the RecoverAll Total Nucleic Acid Isolation Kit for FFPE (Invitrogen, Burlington, ON, Canada). Slight modifications were made to the manufacturer’s protocol to maximize the RNA yield. Specifically, the initial centrifugation to submerge the tissue in the melting buffer was eliminated. Samples were then incubated at 65 °C for 10 min, instead of 72 °C as suggested in the manufacturer’s protocol. Incubation with Proteinase K was done at 60 °C overnight. Lastly, the final elution step was performed twice. The eluted product was then treated with the DNase I and DNase I Buffer included in the RNA extraction kit. The RNA quantity and quality were measured using the Thermo Scientific Nanodrop 2000 (Thermo Fisher Scientific, Waltham, MA, USA). Only RNA with a 260/280 ratio of 1.8–2.1 was used for further analysis. The RNA was stored at −80 °C until being used for cDNA synthesis and amplification. 

### 2.4. Reverse Transcriptase Polymerase Chain Reaction (RT–PCR) for the Optimization Step

For each sample, we converted 10 µL of the RNA into cDNA using the Superscript First-Strand Synthesis System kits (Invitrogen). Following cDNA conversion, PCR (45 cycles) and gel electrophoresis were performed as described previously [10]. Glyceraldehyde-3-phosphate dehydrogenase (GAPDH), a housekeeping gene, was used as an internal control.

### 2.5. cDNA Synthesis and Amplification for NanoString

Amplification of our cDNA products was performed using the nCounter Low RNA Input Kit (NanoString, Seattle, WA, USA) based on the manufacturer’s protocol. Briefly, 4 µL of RNA was used for the cDNA conversion, and the PanCancer Pathways primer pools were used for the cDNA amplification. The cDNA was then amplified for 10 cycles as recommended by the manufacturer for FFPE tissues, then incubated at 95 °C for 2 min. For each sample, a total of 7.5 µL of the amplified cDNA products was produced. The samples were stored at −80 °C until an analysis was conducted with the nCounter platform from NanoString.

### 2.6. NanoString Analysis

All samples were processed by our Institutional Core Facility for NanoString nCounter Analysis. A total of 5 µL of the amplified cDNA product was used for the analysis and the nCounter PanCancer Pathways Panel was used. This panel included 724 cancer-related genes and 60 reference genes (including positive controls, negative controls and housekeeping genes). The panel was designed by NanoString, and all of the included cancer-related gene targets are known to be implicated in at least one cancer pathway. The list of the cancer pathways for each gene target was provided by NanoString. All data normalization was performed by the nSolver 4.0 software (NanoString, Seattle, WA, USA), as recommended by the manufacturer. The genes with the highest differential expression between the tumor MF and non-tumor MF groups were identified by calculating the means of both groups, and dividing the means of the tumor MF group by the non-tumor MF group to obtain the tumor/non-tumor MF ratio. The tumor/non-tumor MF ratios were ranked in descending order, and the highest 30 and lowest 30 genes were identified as the top most upregulated and downregulated genes, respectively, in tumor MF genes. The p-values for the difference of means between the tumor and non-tumor groups for these genes were calculated using Student’s *t*-test. The nSolver 4.0 software was employed to create the heat maps and to perform the Differential Expression analysis. The differential expression analysis identifies the genes with the most statistically significant increased or decreased gene expression (i.e., the most statistically significant log2 fold change) between the tumor and non-tumor groups. The p-values for the differential expression analysis were calculated by the nSolver 4.0 software using a Wald test. 

### 2.7. Immunohistochemistry

Tissue sections with a thickness of 4 µm, derived from FFPE tissue blocks, were used for this study. Deparaffinization was performed by submerging the slides in xylene. After rehydration in graded alcohols, the tissue sections were microwaved in a pressure cooker in citrate buffer, pH 6.0, for 10–15 min for antigen retrieval. The slides were then blocked with fish gelatin and incubated with the primary antibody at 4 °C overnight. Anti-HMGA1 (1:200, Elabscience, Houston, TX, USA; #17331) and anti-SHP2 (1:75, Elabscience, #14341) antibodies were used. Following an incubation with the primary antibodies, the slides were blocked in 3% hydrogen peroxide prior to a one-hour incubation with Dako EnVision+ System HRP Labelled Polymer secondary antibody (Agilent, Santa Clara, CA, USA) at room temperature. The slides were developed in Dako DAB+ Chromagen (Agilent) and were then placed in 1% copper sulfate. The slides were stained with hematoxylin, placed in lithium carbonate for 2 min, and then dehydrated through graded alcohols and xylene. The slides were then coverslipped with Permount Mounting Medium (Thermo Fisher Scientific). The slides were scored by a pathologist. Cases were scored as >50%, representing positive staining, if >50% of the MF cells had stronger staining than the surrounding basal epithelial cells. Cases were scored as ≤50% if 50% or less of the MF cells had stronger staining than the surrounding basal epithelial cells. 

## 3. Results

### 3.1. Optimization of a Protocol to Generate Sufficient cDNA for NanoString 

Based on the NanoString protocol, 10 ng of RNA is the minimum quantity for this analysis, provided that the Low RNA Input Kit is used in the amplification step. Since we identified two prior publications in which RNA extracted from 60–200 singly dissected neurons/astrocytes from frozen tissue sections was sufficient for NanoString [11,12], we chose to microdissect 700–800 single cells from FFPE tissue sections. For this optimization step, we employed FFPE cell blocks containing SK-N-SH, a neuroblastoma cell line [13]. After six independent runs of single-cell dissection and RNA extraction, we found that 700–800 microdissected single cells, typically coming from 5–6 tissue sections, yielded an RNA concentration of 3.9–7.8 ng/µL (median 5.5 ng/µL; SD +/− 1.5 ng/µL). Based on the manufacturer’s protocol for RNA amplification using the Low RNA Input Kit, a maximum of 4.0 µL of RNA sample can be added to the reaction. Thus, a median of 22.0 ng of RNA was used for amplification. Using 4 µL from one of the six generated RNA samples (6.1 ng/µL), we obtained 7.5 µL of cDNA product with a concentration of 2132.6 ng/µL.

To provide evidence that the RNA extracted from our singly extracted SK-N-SH cells was intact, we performed a PCR to amplify the GAPDH gene. As shown in Supplementary Figure S1, a GAPDH band was readily detectable in the RNA sample derived from the singly dissected SK-N-SH cells. Similar GAPDH bands were also detectable in the RNA samples extracted from paraffin curls of the cell blocks as well as from fresh SK-N-SH cells.

### 3.2. Generation of cDNA Samples from Singly Dissected MF Cells

We then studied a total of ten skin biopsy samples from six patients. Two of the six patients had two biopsy samples obtained at different locations, one had three biopsy samples obtained at different locations, and four patients had only one sample. At the time of the skin biopsy, six samples were in the patch/plaque stage and four samples were in the tumor stage. Using our optimized protocol described above, we performed single-cell microdissection of 700–800 MF cells. For the six cases of patch/plaque MF, only lymphoid cells present in the epidermis were captured. For the four cases of tumor MF, MF cells in the dermal tumors were selected. The cDNA concentrations after the Low RNA Input Kit amplification for these 10 samples had a median of 2611.5 ng/µL +/− 637.1 ng/µL. A total of 7.5 µL of amplified cDNA were consistently obtained for each sample. Again, based on the manufacturer’s protocol, 5 µL (i.e., a median of 13 µg of cDNA) from each sample was used for the NanoString assay.

### 3.3. NanoString Analysis

There were 724 cancer-related genes and 60 reference genes (including positive controls, negative controls and housekeeping genes) included in the nCounter PanCancer Pathways Panel. The NanoString nSolver software did not show any housekeeping normalization flags, suggesting that our RNA samples were sufficient for the analysis. Additionally, the nSolver software did not show any positive control flags, indicating that the NanoString assay has a high sensitivity in quantifying gene expression in our samples.

**Figure 1:**
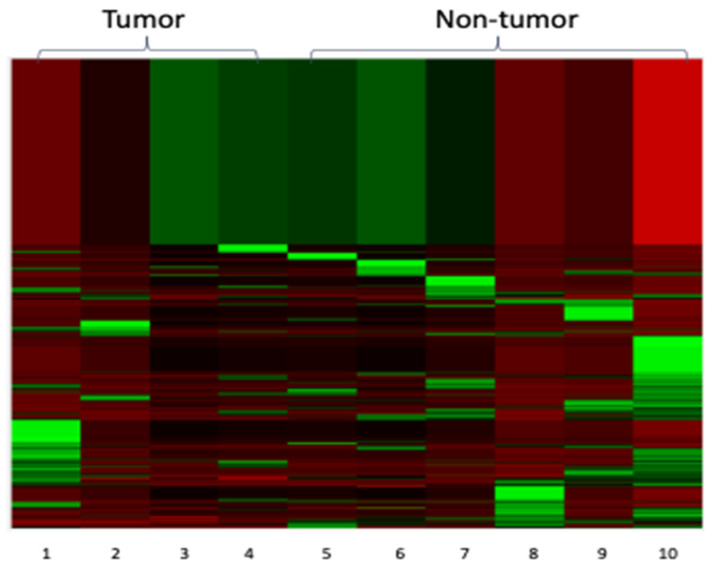
Heat map of all 724 cancer-related genes in the 10 MF patient samples. The NanoString nSolver Analysis Software used hierarchical clustering to group samples and genes together based on relatedness. Red shading indicates low gene expression relative to the average expression, black shading indicates average expression and green shading indicates high gene expression relative to the average expression. All 4 tumor MF samples were clustered on the left of the panel and all 6 non-tumor MF samples were clustered on the right of the panel.

Normalization of the collected data against the 60 reference genes was performed using the NanoString nSolver analysis software. A heat map illustrating clustered samples and genes based on their relatedness (i.e., un-supervised) was generated (Figure 1). We noted that all four tumor samples were localized to the left side of the panel (i.e., columns 1–4) whereas the six patch/plaque MF samples were found on the right side of the panel (columns 5–10). 

**Figure 2:**
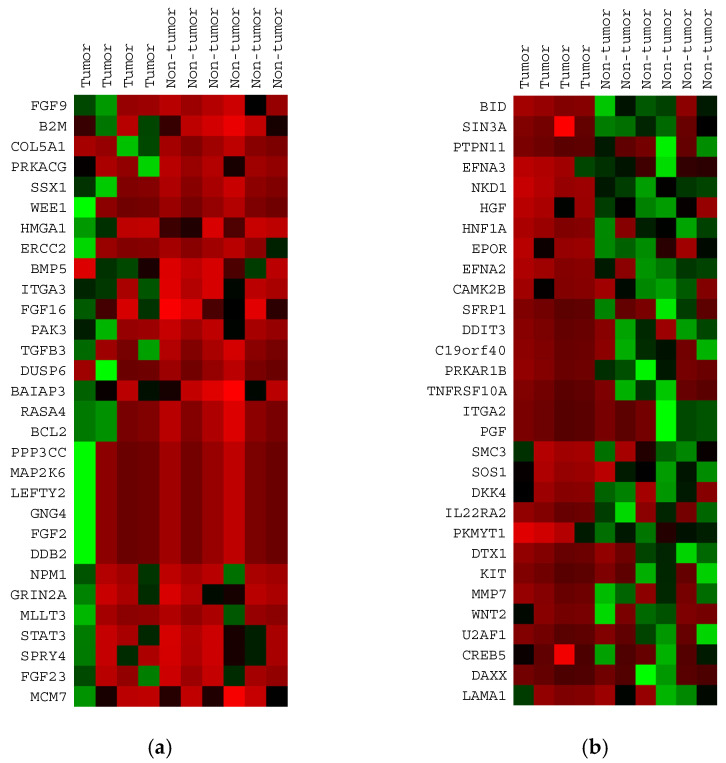
(**a**) Heat map of the 10 MF samples generated using the 30 most upregulated genes in tumor MF (i.e., the genes with the highest tumor/non-tumor MF ratio). The 6 non-tumor samples show a pattern of red shading indicating low gene expression, while the 4 tumor samples show a pattern of green shading indicating high gene expression. (**b**) Heat map off the 10 MF samples generated using the 30 most downregulated genes in MF. The 6 non-tumor samples show a pattern of green shading indicating high gene expression, while the 4 tumor samples show a pattern of red shading indicating low gene expression.

We then determined which of the 724 cancer-related genes were most significantly different between non-tumor and tumor MF. For each of these genes, we calculated the mean for each group, and we ranked the genes based on the descending tumor/non-tumor MF ratio. The top 30 upregulated genes in tumor MF are summarized in Table 1 and the top 30 downregulated genes in tumor MF are summarized in Table 2. The top 30 upregulated genes in tumor MF had a tumor/non-tumor MF ratio of >1.34, and the top 30 downregulated genes in tumor MF had a tumor/non-tumor MF ratio of <0.60. As shown in Figure 2, heat maps generated using these top 30 upregulated and downregulated genes showed a clearer separation between the tumor and the non-tumor groups compared to that illustrated in Figure 1.

The validity of these gene sets was further tested using the differential expression analysis from the NanoString nSolver software, which identified the top 30 genes showing differences between the tumor and non-tumor MF groups with the highest statistical significance. As shown in Table 3, three genes, including *B2M*, *FGF9* and *HMGA1*, were also found in the 30 upregulated (in tumor MF) gene list. Twenty-two genes, including *SIN3A*, *BID*, *HGF*, *EPOR*, *CAMK2B*, *CREB5*, *PTPN11*, *NKD1*, *HNF1A*, *EFNA2*, *SMC3*, *SFRP1*, *PKMYT1*, *C19orf40*, *DDIT3*, *SOS1*, *DKK4*, *PRKAR1B*, *TNFRSF10A*, *LAMA1*, *ITGA2* and *PGF* were found in the 30 downregulated (in tumor MF) gene list.

As shown in Table 4, we summarized the cancer pathways with which the 30 upregulated and 30 downregulated genes are known to be associated. The known cancer pathways of all gene targets were provided in a list from NanoString. The pathways that are most represented by these identified gene targets are the PI3K pathway (18/60), the RAS pathway (16/60), the cell cycle/apoptosis pathway (12/60), the MAPK pathway (12/60) and the Wnt pathway (8/60).

### 3.4. Target Validation Using Immunohistochemistry

We selected two gene targets for further validation using immunohistochemistry, namely *HMGA1* and *PTPN11* (encodes SHP2), both of which were identified as one of the highest upregulated or downregulated genes based on the analysis of the non-tumor MF/tumor MF ratios as well as being in the top 30 most statistically significant genes in the differential expression analysis. Other criteria for their inclusion for the validation studies are related to the availability of commercial antibodies and their known oncogenic or tumor suppressor functions found in other cancer types. *HMGA1*, known to be oncogenic in other cancer types [14,15,16], was found to be upregulated in tumor MF. *PTPN11*, encoding SHP2, which has been found to be a tumor suppressor in several cancer models [17,18,19], was found to be downregulated in tumor MF. 

**Figure 3:**
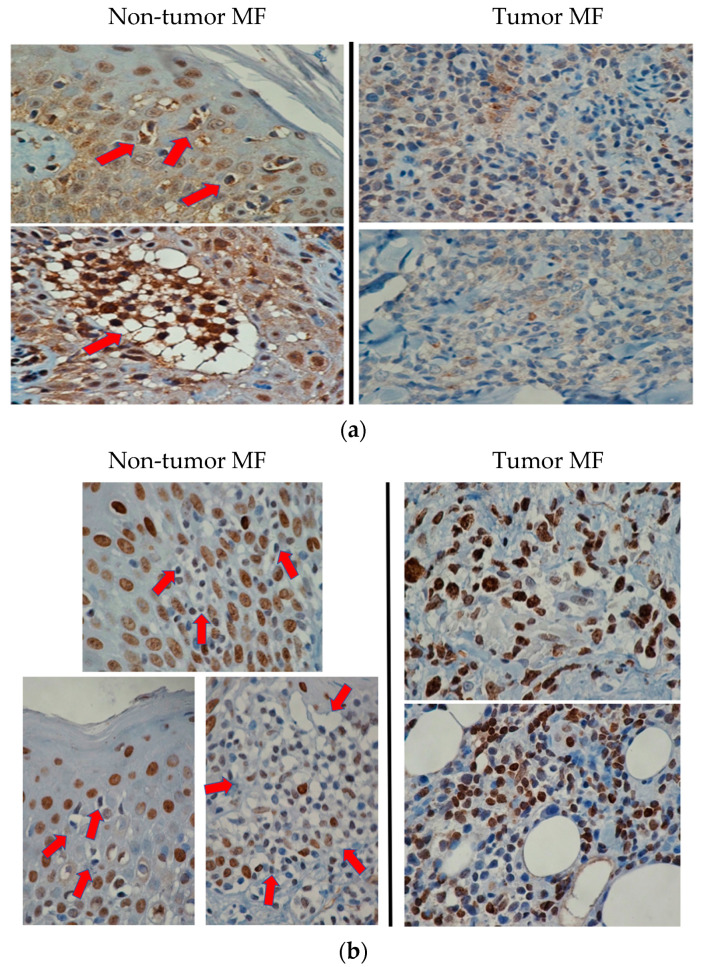
Immunohistochemical validation of SHP2 and HMGA1. (**a**) Immunostaining of SHP2 showed strong cytoplasmic staining of epidermotropic MF cells (highlighted by red arrows) in two non-tumor MF cases (left panel). The strong staining of the basal epithelial cells served as the internal reference. In comparison, SHP2 was weak or undetectable in most cells present in the MF tumors (right panel) (400×). (**b**) MF cells in the epidermis in three non-tumor cases (highlighted by red arrows) showed no appreciable HMGA1 nuclear expression (left panel). The basal epithelial cells served as the internal reference. Neoplastic cells in MF tumors showed strong HMGA1 nuclear staining (right panel) (400×).

As summarized in Table 5, a total of 11 samples (6 non-tumor MF and 5 tumor MF) derived from 6 patients were included in the immunohistochemical validation. Three patients (#1, 2 and 4) had both non-tumor MF and tumor MF samples. For HMGA1 (nuclear staining pattern) and SHP2 (cytoplasmic staining pattern), positivity was assessed when there were >50% MF cells showing staining greater than that of basal epithelial cells. In non-tumor MF cases, immunostaining was evaluated only for lymphoid cells in the epidermis. In tumor MF cases, immunostaining was evaluated on lymphoid cells in the dermis where MF clusters or sheets were found. As summarized in Table 5, HMGA1 was positive in six of six non-tumor MF samples and negative in five of five tumor MF samples. Similarly, SHP2 was negative in six of six non-tumor MF samples and positive in five of five tumor MF samples. These differences are statistically significant (Fisher’s exact test, *p* = 0.0022). Images of HMGA1 and SHP2 staining in MF non-tumor and tumor cases are shown in Figure 3.

## 4. Discussion

One of the major advantages of analyzing samples derived from single-cell microdissection is that cells of interest are specifically targeted. Thus, contamination or dilution with ‘unwanted’ cells can be minimized and accuracy can be improved. Single-cell microdissection is well-suited for gene expression profiling (GEP) of MF cells, since these neoplastic cells are often surrounded by an abundance of benign epithelial cells, stromal cells and reactive immune cells. Nonetheless, GEP coupled with single-cell microdissection is technically difficult and rarely done. In our literature search, we were able to identify only two studies employing this protocol [11,12]. In both studies, small numbers (60–200) of singly dissected human or mouse neurons/astrocytes were harvested from frozen tissue sections, the extracted RNA was amplified using multiplexed target enrichment amplification and the generated cDNA samples were analyzed using NanoString. With this background, we asked if single-cell microdissection/GEP is feasible if FFPE tissues are used. If successful, this protocol can be used to study human diseases for which FFPE tissues are far more available than frozen tissues.

Thus, the first objective of this study is to develop/optimize a protocol by which sufficient RNA can be extracted from single-cell microdissection using FFPE tissues. Our studies have shown that RNA extracted from 700–800 MF cells, after being subjected to multiplexed target enrichment amplification (i.e., Low RNA Input Kit), was sufficient for NanoString analysis. In our experience, this protocol can produce GEP results consistently, as we were able to obtain results in all the tissue blocks we processed. The validity of our protocol is supported by the fact that our results passed all of the NanoString built-in checkpoints. This protocol can be highly useful in studying other types of tumors in which the neoplastic cell population represents only a small component of the tumor and/or the neoplastic cells are often surrounded by abundant benign cells. T-cell/histiocyte-rich B-cell lymphomas, Hodgkin lymphomas and the inflammatory variants of various sarcomas/carcinomas likely fall into this category.

Despite the relatively small sample size, our single-cell microdissection/NanoString analysis was effective in differentiating tumor MF from non-tumor MF. Our observation that tumor MF cases (n = 4) could be separated from non-tumor MF cases (n = 6) using un-supervised analysis suggests that our method of cell selection is valid. Thus, in non-tumor MF cases, only lymphoid cells in the epidermis (i.e., epidermotropic) were selected. This approach will minimize the likelihood of including benign lymphocytes, which can extensively infiltrate the dermis. In tumor MF cases, cells were selected from relatively well-defined tumorous regions in the dermis, where MF cells form clusters and their cytological abnormalities can be readily appreciated.

The second objective of this study is to decipher the molecular events underlying the progression from non-tumor MF to tumor MF. In this regard, we could not identify any similar studies in the literature. We identified that the PI3K and RAS pathways are the two pathways most implicated. Interestingly, we noticed that many of the gene targets identified overlap between these two pathways, such as *FGF9*, *FGF16*, *FGF2*, *GNG4*, *FGF23*, *EFNA3*, *EFNA2*, *HGF*, *PGF*, *SOS1* and *KIT*, suggesting that the cross-talks between the PI3K and RAS pathways might be important in promoting progression to the tumor stage. In this regard, it has been shown that, in certain cancer models, activated RAS is required for the full execution of the oncogenic activity of PI3K [20]. A similar scenario may occur in the disease progression of MF, which requires significant deregulations of both signaling pathways. This requirement may partly explain why progression to tumor-stage MF is a relatively infrequent occurrence.

To further validate the genes identified with our GEP studies, we performed immunohistochemical analysis. HMGA1 (i.e., upregulated in tumor MF) and PTPN11/SHP2 (i.e., downregulated in MF) were selected. HMGA1 overexpression has been identified in a number of cancer types, and this abnormality has been associated with a poor prognosis in patients with gliomas, pancreatic cancer and lung cancer [15,16]. HMGA1 has been shown to contribute to the maintenance of cancer stem cell populations and promote cell proliferation, angiogenesis and anchorage-independent growth [14,15,21]. SHP2, encoded by PTPN11, has been shown to play tumor-suppressor roles in a number of cancer models such as hepatocellular carcinomas [17,18,19]. In these studies, SHP2 has been shown to inhibit the JAK-STAT pathway, which is known to be activated in many cancer types [22,23]. In keeping with these concepts, we observed a downregulation of SHP2 and an upregulation of STAT3 in tumor MF. These observations correlate well with another MF study, in which STAT3 activation was found to be higher in advanced stages of MF compared to early MF stages [24].

In conclusion, the results from this study have provided a proof-of-concept that RNA samples derived from single-cell microdissection applied to archival tumor samples can be used consistently for GEP. While this study contains limited MF cases, we have shown that our protocol is feasible to study MF, and we hope that it will stimulate future MF studies using this methodology. We believe that this protocol can be valuable in studying other cancer cells that are surrounded by abundant benign cells and/or cells irrelevant to the study. Our results have also highlighted specific cancer pathways and gene targets that might be important in mediating the progression of MF to the tumor stage.

## Figures and Tables

**Table 1:** Summary of the 30 genes most upregulated in tumor MF based on the tumor/non-tumor MF ratio.

Gene Name	Mean of Non-Tumor MF	Mean of Tumor MF	SD of Non-Tumor MF	SD of Tumor MF	*p*-Value	Tumor/Non-Tumor
*FGF9*	1.60	3.12	0.63	1.97	0.22	1.95
*B2M*	1.83	3.54	0.77	1.79	0.15	1.94
*COL5A1*	1.36	2.55	0.13	1.52	0.21	1.88
*PRKACG*	1.55	2.89	0.38	2.16	0.31	1.86
*SSX1*	1.36	2.45	0.13	1.24	0.18	1.80
*WEE1*	1.36	2.42	0.13	1.93	0.35	1.78
*HMGA1*	2.01	3.44	0.68	2.47	0.33	1.71
*ERCC2*	1.61	2.75	0.69	2.59	0.45	1.71
*BMP5*	2.03	3.24	1.16	1.42	0.21	1.60
*ITGA3*	1.55	2.48	0.38	0.67	0.06	1.60
*FGF16*	2.22	3.51	0.94	1.72	0.24	1.59
*PAK3*	1.55	2.45	0.38	1.24	0.25	1.58
*TGFB3*	1.36	2.13	0.13	0.82	0.15	1.57
*DUSP6*	1.36	2.11	0.13	1.34	0.34	1.56
*BAIAP3*	1.81	2.81	0.72	1.02	0.15	1.55
*BCL2*	1.36	2.10	0.13	0.69	0.12	1.55
*RASA4*	1.36	2.10	0.13	0.69	0.12	1.55
*DDB2*	1.36	2.09	0.13	1.27	0.33	1.54
*FGF2*	1.36	2.09	0.13	1.27	0.33	1.54
*GNG4*	1.36	2.09	0.13	1.27	0.33	1.54
*LEFTY2*	1.36	2.09	0.13	1.27	0.33	1.54
*MAP2K6*	1.36	2.09	0.13	1.27	0.33	1.54
*PPP3CC*	1.36	2.09	0.13	1.27	0.33	1.54
*NPM1*	2.13	3.17	1.78	2.03	0.44	1.49
*GRIN2A*	1.77	2.47	0.56	1.26	0.36	1.39
*MLLT3*	1.74	2.42	0.84	1.93	0.55	1.39
*SPRY4*	1.79	2.47	0.64	1.26	0.38	1.38
*STAT3*	1.79	2.47	0.64	1.26	0.38	1.38
*FGF23*	1.55	2.13	0.38	0.82	0.26	1.38
*MCM7*	2.05	2.76	0.79	1.80	0.50	1.35

**Table 2:** Summary of the 30 genes most downregulated in tumor MF based on the tumor/non-tumor MF ratio.

Gene Name	Mean of Non-Tumor MF	Mean of Tumor MF	SD of Non-Tumor MF	SD of Tumor MF	*p*-Value	Tumor/Non-Tumor
*BID*	3.54	1.43	1.67	0.09	0.03	0.40
*SIN3A*	5.77	2.48	1.89	0.67	0.01	0.43
*PTPN11*	3.04	1.43	2.25	0.09	0.14	0.47
*NKD1*	2.94	1.43	0.57	0.09	0.00	0.48
*EFNA3*	5.32	2.55	4.29	2.31	0.22	0.48
*EFNA2*	2.89	1.43	0.86	0.09	0.01	0.49
*EPOR*	3.60	1.77	1.60	0.65	0.04	0.49
*HNF1A*	2.93	1.43	1.03	0.09	0.02	0.49
*HGF*	3.70	1.80	1.61	0.80	0.04	0.49
*CAMK2B*	3.51	1.77	1.91	0.65	0.08	0.50
*DDIT3*	2.79	1.43	1.39	0.09	0.06	0.51
*SFRP1*	2.80	1.43	1.77	0.09	0.12	0.51
*C19orf40*	2.76	1.43	1.40	0.09	0.07	0.52
*PRKAR1B*	2.67	1.43	1.42	0.09	0.08	0.53
*ITGA2*	2.62	1.43	1.72	0.09	0.15	0.54
*PGF*	2.62	1.43	1.72	0.09	0.15	0.54
*TNFRSF10A*	2.64	1.43	1.52	0.09	0.11	0.54
*SMC3*	3.77	2.09	1.65	1.27	0.11	0.55
*SOS1*	3.14	1.76	1.25	0.60	0.05	0.56
*KIT*	2.49	1.43	1.41	0.09	0.12	0.57
*DTX1*	2.50	1.43	1.09	0.09	0.06	0.57
*PKMYT1*	3.16	1.80	0.65	0.80	0.03	0.57
*IL22RA2*	2.50	1.43	1.13	0.09	0.07	0.57
*DKK4*	3.09	1.76	1.43	0.60	0.08	0.57
*U2AF1*	2.47	1.43	1.32	0.09	0.11	0.58
*WNT2*	3.05	1.76	2.02	0.60	0.19	0.58
*MMP7*	2.48	1.43	0.99	0.09	0.05	0.58
*LAMA1*	3.53	2.09	2.31	1.27	0.24	0.59
*DAXX*	2.41	1.43	1.64	0.09	0.20	0.59
*CREB5*	4.79	2.81	2.51	1.02	0.13	0.59

**Table 3:** Differential expression analysis revealed the 30 gene targets with the highest statistically significant upregulated (3 genes) or downregulated (27 genes) gene expression in tumor MF groups compared to non-tumor MF groups. These 30 gene targets substantially overlap with the top upregulated and downregulated genes identified using the tumor/non-tumor MF ratios, with 25 of these 30 genes being listed in Table 1 or Table 2.

Downregulated in Tumor MF			Upregulated in Tumor MF		
Gene	Log2 Fold Change	*p*-Value	Gene	Log2 Fold Change	*p*-Value
*SIN3A*	−1.31	0.0728	B2M	0.908	0.232
*BID*	−1.41	0.127	FGF9	0.945	0.241
*HGF*	−1.18	0.156	HMGA1	0.727	0.318
RHOA *	−0.601	0.159			
*EPOR*	−1.1	0.187			
*CAMK2B*	−1.09	0.189			
*CREB5*	−0.866	0.196			
*PTPN11*	−1.18	0.201			
*NKD1*	−1.12	0.221			
*HNF1A*	−1.12	0.222			
*EFNA2*	−1.12	0.222			
*SMC3*	−0.925	0.226			
*SFRP1*	−1.1	0.231			
*PKMYT1*	−0.905	0.273			
*C19orf40*	−1	0.273			
*DDIT3*	−1	0.273			
*SOS1*	−0.902	0.275			
*DKK4*	−0.901	0.275			
*PRKAR1B*	−0.997	0.276			
*TNFRSF10A*	−0.995	0.277			
WNT2 *	−0.892	0.281			
*LAMA1*	−0.819	0.286			
*ITGA2*	−0.978	0.288			
*PGF*	−0.978	0.288			
MEN1 *	−0.795	0.335			
GPC4 *	−0.792	0.337			
GLI3 *	−0.791	0.338			

* *RHOA*, *WNT2*, *MEN1*, *GPC4* and *GLI3* were not identified in the gene lists in Table 1 and Table 2.

**Table 4:** Cancer pathways implicated in gene targets differentially expressed between non-tumor and tumor MF.

Pathway	Upregulated Genes	Downregulated Genes
PI3K	*FGF9*, *COL5A1*, *ITGA3*, *FGF16*, *BCL2*, *FGF2*, *GNG4*, *FGF23*	*EFNA3*, *EFNA2*, *EPOR*, *HGF*, *ITGA2*, *PGF*, *SOS1*, *KIT*, *LAMA1*, *CREB5*
RAS	*FGF9*, *PRKACG*, *FGF16*, *PAK3*, *RASA4*, *FGF2*, *GNG4*, *GRIN2A*, *FGF23*	*PTPN11*, *EFNA3*, *EFNA2*, *HGF*, *PGF*, *SOS1*, *KIT*
Cell Cycle and Apoptosis	*PRKACG*, *WEE1*, *TGFB3*, *BCL2*, *DDB2*, *PPP3CC*, *MCM7*	*BID*, *PRKAR1B*, *TNFRSF10A*, *SMC3*, *PKMTY1*
MAPK	*FGF9*, *PRKACG*, *FGF16*, *TGFB3*, *DUSP6*, *FGF2*, *MAP2K6*, *PPP3CC*, *FGF23*	*DDIT3*, *SOS1*, *DAXX*
Wnt	*PRKACG*, *PPP3CC*	*NKD1*, *CAMK2B*, *SFRP1*, *DKK4*, *WNT2*, *MMP7*
Driver Gene	*B2M*, *BCL2*, *NPM1*	*PTPN11*, *HNF1A*, *KIT*, *U2AF1*, *DAXX*
JAK-STAT	*SPRY4*, *STAT3*	*PTPN11*, *EPOR*, *SOS1*, *IL22RA2*
Transcriptional Regulation	*SSX1*, *DUSP6*, *BAIAP3*, *MLLT3*	*SIN3A*, *DDIT3*
TGF-Beta	*BMP5*, *TGFB3*, *LEFTY2*	
DNA Repair	*ERCC2*	*C19orf40*
Hedge Hog	*PRKACG*	*WNT2*
Chromatin Modification	*HMGA1*	
Notch		*DTX1*

**Table 5:** Immunohistochemical analysis of MF patients showed SHP2 protein expression downregulated in tumor MF cases and HMGA1 protein expression upregulated in tumor MF cases.

Patient No.	Non-Tumor MF or Tumor MF	NanoString Data	SHP2	HMGA1
1	non-tumor	Yes	>50%	≤50%
1	tumor	Yes	≤50%	>50%
1	tumor	Yes	≤50%	>50%
2	non-tumor	Yes	>50%	≤50%
2	tumor	Yes	≤50%	>50%
3	tumor	Yes	≤50%	>50%
4	non-tumor	No	>50%	≤50%
4	tumor	No	≤50%	>50%
5	non-tumor	No	>50%	≤50%
5	non-tumor	No	>50%	≤50%
6	non-tumor	No	>50%	≤50%

## Data Availability

The data presented in this study are openly available at https://doi.org/10.3390/xxxxx.

## Supplementary Materials

The following are available online at https://www.mdpi.com/article/10.3390/cells10113190/s1, Table S1: Characteristics of the patients used in the NanoString and/or immunohistochemical analysis. Figure S1: RT-PCR was performed to detect *GAPDH*.

## References

[1] Swerdlow S.H., Campo E., Harris N.L., Jaffe E.S., Pileri S.A., Stein H., Thiele J. (2017). Mycosis Fungoides. WHO Classification of Hematological Malignancy.

[2] Vergier B., de Muret A., Beylot-Barry M., Vaillant L., Ekouevi D., Chene G., Carlotti A., Franck N., Dechelotte P., Souteyrand P. (2000). Transformation of mycosis fungoides: Clinicopathological and prognostic features of 45 cases. French study group of cutaneous lymphomas. Blood.

[3] Dmitrovsky E., Matthews M.J., Bunn P.A., Schechter G.P., Makuch R.W., Winkler C.F., Eddy J., Sausville E.A., Ihde D.C. (1987). Cytologic transformation in cutaneous T cell lymphoma: A clinicopathologic entity associated with poor prognosis. J. Clin. Oncol..

[4] van Santen S., Roach R.E., van Doorn R., Horváth B., Bruijn M.S., Sanders C.J., de Pooter J.C., van Rossum M.M., de Haas E.R., Veraart J.C. (2016). Clinical Staging and Prognostic Factors in Folliculotropic Mycosis Fungoides. JAMA Dermatol..

[5] Li L., Lei Q., Zhang S., Kong L., Qin B. (2017). Screening and identification of key biomarkers in hepatocellular carcinoma: Evidence from bioinformatic analysis. Oncol. Rep..

[6] Morais-Rodrigues F., Silverio-Machado R., Kato R.B., Rodrigues D.L.N., Valdez-Baez J., Fonseca V., San E.J., Gomes L.G.R., dos Santos R.G., Vinicius Canário Viana M. (2020). Analysis of the microarray gene expression for breast cancer progression after the application modified logistic regression. Gene.

[7] Varambally S., Dhanasekaran S.M., Zhou M., Barrette T.R., Kumar-Sinha C., Sanda M.G., Ghosh D., Pienta K.J., Sewalt R.G., Otte A.P. (2002). The polycomb group protein EZH2 is involved in progression of prostate cancer. Nature.

[8] Hashikawa K., Yasumoto S., Nakashima K., Arakawa F., Kiyasu J., Kimura Y., Saruta H., Nakama T., Yasuda K., Tashiro K. (2014). Microarray analysis of gene expression by microdissected epidermis and dermis in mycosis fungoides and adult T-cell leukemia/lymphoma. Int. J. Oncol..

[9] Zhang Y., Wang Y., Yu R., Huang Y., Su M., Xiao C., Martinka M., Dutz J.P., Zhang X., Zheng Z. (2012). Molecular Markers of Early-Stage Mycosis Fungoides. J. Investig. Dermatol..

[10] Haque M., Li J., Huang Y., Almowaled M., Barger C.J., Karpf A.R., Wang P., Chen W., Turner S.D., Lai R. (2019). NPM-ALK Is a Key Regulator of the Oncoprotein FOXM1 in ALK-Positive Anaplastic Large Cell Lymphoma. Cancers.

[11] Tagliafierro L., Bonawitz K., Glenn O.C., Chiba-Falek O. (2016). Gene Expression Analysis of Neurons and Astrocytes Isolated by Laser Capture Microdissection from Frozen Human Brain Tissues. Front. Mol. Neurosci..

[12] Katz I.K., Lamprecht R. (2015). Fear conditioning leads to alteration in specific genes expression in cortical and thalamic neurons that project to the lateral amygdala. J. Neurochem..

[13] Biedler J.L., Helson L., Spengler B.A. (1973). Morphology and growth, tumorigenicity, and cytogenetics of human neuroblastoma cells in continuous culture. Cancer Res..

[14] Huso T.H., Resar L.M. (2014). The high mobility group A1 molecular switch: Turning on cancer-can we turn it off?. Expert Opin. Ther. Targets.

[15] Schuldenfrei A., Belton A., Kowalski J., Talbot J., Conover C., Di Cello F., Poh W., Tsai H., Shah S.N., Huso T.H. (2011). HMGA1 drives stem cell, inflammatory pathway, and cell cycle progression genes during lymphoid tumorigenesis. BMC Genom..

[16] Wang Y., Hu L., Zheng Y., Guo L. (2019). HMGA1 in cancer: Cancer classification by location. J. Cell. Mol. Med..

[17] Bard-Chapeau E., Li S., Ding J., Zhang S., Zhu H., Princen F., Fang D., Han T., Bailly-Maitre B., Poli V. (2011). Ptpn11/Shp2 Acts as a Tumor Suppressor in Hepatocellular Carcinogenesis. Cancer Cell.

[18] Jiang C., Hu F., Tai Y., Du J., Mao B., Yuan Z., Wang Y., Wei L. (2012). The tumor suppressor role of Src homology phosphotyrosine phosphatase 2 in hepatocellular carcinoma. J. Cancer Res. Clin. Oncol..

[19] Seif F., Khoshmirsafa M., Aazami H., Mohsenzadegan M., Sedighi G., Bahar M. (2017). The role of JAK-STAT signaling pathway and its regulators in the fate of T helper cells. Cell Commun. Signal..

[20] Rampias T., Giagini A., Siolos S., Matsuzaki H., Sasaki C., Scorilas A., Psyrri A. (2014). Crosstalk and Cetuximab Resistance in Head and Neck Squamous Cell Carcinoma. Clin. Cancer Res..

[21] Pang B., Fan H., Zhang I.Y., Liu B., Feng B., Meng L., Zhang R., Sadeghi S., Guo H., Pang Q. (2012). HMGA1 expression in human gliomas and its correlation with tumor proliferation, invasion and angiogenesis. J. Neurooncol..

[22] Thomas S.J., Snowden J.A., Zeidler M.P., Danson S.J. (2015). The role of JAK/STAT signalling in the pathogenesis, prognosis and treatment of solid tumours. Br. J. Cancer.

[23] Groner B., von Manstein V. (2017). Jak Stat signaling and cancer: Opportunities, benefits and side effects of targeted inhibition. Mol. Cell. Endocrinol..

[24] Pérez C., Mondéjar R., García-díaz N., Cereceda L., León A., Montes S., Durán Vian C., Pérez Paredes M.G., González-morán A., Miguel V. (2020). Advanced-stage mycosis fungoides: Role of the signal transducer and activator of transcription 3, nuclear factor-κB and nuclear factor of activated T cells pathways. Br. J. Derm..

