# Supplementary material for: Gene Expression Profiling of Mycosis Fungoides in Early and Tumor Stage—A Proof-of-Concept Study Using Laser Capture/Single Cell Microdissection and NanoString Analysis
Source: Cells. 2021 Nov 16;10(11):3190. doi: 10.3390/cells10113190 (PMC8621072; doi:10.3390/cells10113190)
Supplement: Supplementary file 1 [file cells-10-03190-s001.zip › cells-1424457-supplementary.pdf]

Patient No.	Specimen No.	Non-tumor or Tumor MF	NanoString/IHC /Both	Age	Gender	Sites (skin biopsy)
1	MF-1	Non-tumor	Both	82	M	Right upper back
	MF-2	Tumor	Both			Right posterior arm
	MF-3	Tumor	Both			Left chest wall
2	MF-4	Non-tumor	Both	56	M	Right scapula
	MF-5	Tumor	Both			Left scalp
3	MF-6	Tumor	Both	44	F	Frontal scalp
4	MF-7	Non-tumor	IHC	59	M	Left medial chest
	MF-8	Tumor	IHC			Left lateral chest
5	MF-9	Non-tumor	IHC	44	F	Left medial foot
	MF-10	Non-tumor	IHC			Right lower abdomen
6	MF-11	Non-tumor	IHC	76	M	Right posterior thigh
7	MF-12	Non-tumor	Nanostring	56	F	Right upper back
	MF-13	Non-tumor	Nanostring			Left lower back
8	MF-14	Non-tumor	Nanostring	42	F	Right flank
9	MF-15	Non-tumor	Nanostring	55	F	Unknown

Supplementary Table S1. Characteristics of the patients used in the NanoString and/or immunohistochemical analysis. All multiple samples from the same patients (#1,2,4,5 and 7) were collected at the same time.

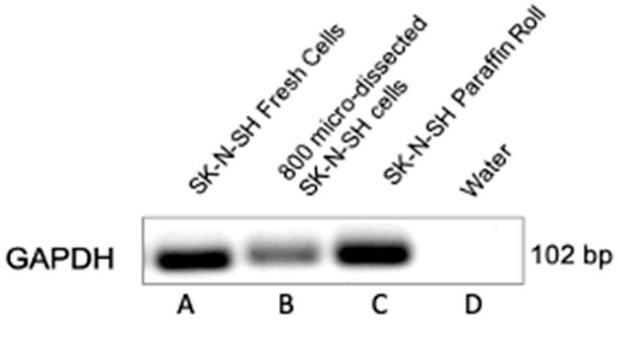

Supplementary Figure S1. RT-PCR was performed to detect *GAPDH*. Lane A was derived from RNA extracted from fresh SK-N-SH cells. Lane B was derived from RNA extracted from 800 single microdissected SK-N-SH cells from FFPE tissue sections. Lane C was derived RNA extracted from curls of a FFPE tissue block containing SK-N-SH. Lane D contained RNase-free water. Our results support that RNA from single-microdissected cells was relatively intact.
